# As the Antarctic Ice Pack Recedes, a Fragile Ecosystem Hangs in the Balance

**DOI:** 10.1371/journal.pbio.0030127

**Published:** 2005-04-12

**Authors:** Liza Gross

## Abstract

Many key species of the Antarctic marine ecosystem--including krill, the backbone of the food chain--depend on the availability of winter sea ice. If global temperatures continue to rise and the winter ice pack continues to recede, this fragile Antarctic marine ecosystem could face collapse

Harrowing tales of starvation and endurance epitomize Antarctica's “heroic age,” when men equipped with little more than fortitude struggled against a landscape seemingly designed to thwart their intentions. A menacing sea ice figures prominently in these improbable survival stories. Daunting to early-20th-century explorers—trapping (and ultimately crushing) Ernest Shackleton's *Endurance* and derailing Robert Scott's 1901 *Discovery* expedition—the seasonal pack ice is the lifeblood of Antarctica's marine ecosystem.

But as winter temperatures continue to climb in the Antarctic, the once-forbidding winter sea ice is starting to deteriorate. The ice pack is forming later and retreating earlier—and it's having a serious impact on the abundance of krill, the backbone of the Antarctic food chain.

“Sea ice is the engine that drives Antarctic ecosystems,” says William Fraser, a principal investigator for the Palmer Long Term Ecological Research program (LTER) and president of Polar Oceans Research Group in Montana. “Many of the key species that govern ecosystem dynamics in the Antarctic have life histories that depend on the availability of winter sea ice. The most important species in most sectors of Antarctica is krill.”

A major food source for Antarctic fish, penguins, pelagic seabirds, seals, and whales, krill (Euphausia superba) look like shrimp, but weigh just a gram as adults and measure about six centimeters long (Figure 1). Norwegian for “whale food,” krill aggregate in super-swarms that can reach a density of 30,000 individuals per square meter, attracting whales, which can eat three tons of krill in a single feeding, and fisheries, which net on average 100,000 metric tons per year.

**Figure 1:**
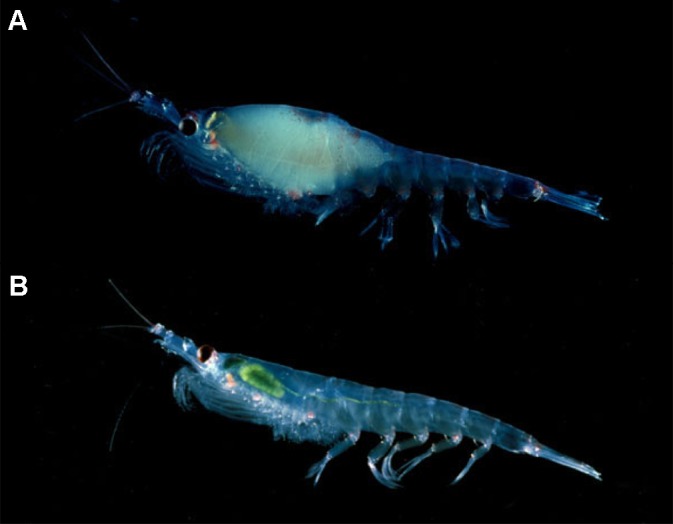
Krill Abundance Has Dropped 80% in 30 Years (A) This gravid female is nearly ready to release her eggs. (B) Krill feed on phytoplankton, indicated by the green color in this specimen's digestive organ. (Images: Langdon Quetin and Robin Ross, researchers at the Marine Science Institute, University of California, Santa Barbara, and funded by the Office of Polar Programs, National Science Foundation)

The waters off the Antarctic Peninsula favor high krill concentrations. “Once you get into extreme environments such as the Southern Ocean, diversity will decrease but the number of individuals will increase because the production can be very high,” says Scott Gallager, a marine biologist at Woods Hole Oceanographic Institution (Woods Hole, Massachusetts, United States).

Production is particularly high along the sea ice edge, he says, because the ice is thinner, which allows more sunlight to penetrate, and because ocean mixing processes along the continental shelf cause an upwelling of nutrient-rich deep water. Increased nutrients support increased primary production along the ice edge. “If ice forms too late,” says Gallager, “you don't get this higher production, which impacts zooplankton populations like the larval krill that graze the under-ice surface, feeding on ice algae. The ice edge is an absolutely critical habitat, a nursery, for larval krill.”

But this krill hotspot is showing some of the most dramatic changes in sea ice extent. Fraser says the western Antarctic Peninsula has registered the “largest increase in temperatures on the planet”—on the order of 6 degrees Celsius—over the past 50 years. This warming trend, he says, has been particularly pronounced during the winter—“crunch time” for many key Antarctic species.

“There was a time when almost every winter experienced very heavy sea ice,” Fraser says, “reaching out into the Drake Passage.” Where once heavy sea ice would form on average four out of every five years, he says, now it forms just one or two years out of five (Box 1). In a typical year, ice starts to form in the coldest regions along the southern coast in late March/early April (austral fall), then works its way up the coast. But late ice years are becoming more common, with ice forming two to three weeks later. “These patterns are completely different from patterns that existed as recently as 30 to 40 or 50 years ago,” says Fraser. “The whole system is becoming unhinged as a result of this enormous warming.”

**Box 1. Coupled Ocean-Atmosphere System Controls Sea Ice Extent**

What are the chances that the western Antarctic Peninsula will start to see more heavy-ice years? Annual winter sea ice extent depends on air-sea interactions between a recently discovered atmospheric phenomenon called the Antarctic Circumpolar Wave (Figure 3) and Circumpolar Deep Water (CDW), which is part of the massive Antarctic Circumpolar Current.

The Antarctic Circumpolar Wave can either bring air up off the Antarctic continent, making the peninsula colder than normal, or down out of the South Pacific or South America, making the peninsula warmer, explains Eileen Hofmann, oceanography professor at the Center for Coastal Physical Oceanography at Old Dominion University (Norfolk, Virginia, United States). “What seems to be happening is that the atmosphere and ocean are transitioning into another state because of the warming in the Antarctic Peninsula, and now there are fewer colder periods,” says Hofmann, who also heads Southern Ocean GLOBEC (Global Oceans Ecosystem Dynamics). “It warms up particularly in the winter because you don't get the strong winds coming up the continent as frequently. And so less sea ice forms in the winter.”

The southern boundary of the Antarctic Circumpolar Current meets the continental shelf along the Antarctic Peninsula. As the current moves back and forth along the shelf, it pumps deep water onto the shelf. “Where you get this upwelling of CDW,” Hofmann says, “it preferentially selects for diatoms, the preferred food for Antarctic krill.” And embryos released around deep water develop faster and hatch at shallower depths, which means they don't have as far to swim to reach the surface, which in turn means less chance of being eaten en route and a better chance of finding food sooner. Once the larvae reach surface waters, the circulation over the continent helps retain them along the western Antarctic Peninsula.

If there's no winter sea ice covering the ocean surface, the momentum from the atmosphere can go into the ocean and enhance mixing, which forces heat up to the surface and prevents sea ice from forming, Hofmann says. “If you raise the temperature to -1.7 degrees Centigrade”—salinity causes ocean water to freeze at −1.82 degrees C—“you get no sea ice. It's that small difference that makes the system very responsive to climate change.”

And melting ice introduces freshwater, which is much lighter than seawater, says Scott Gallager of the Woods Hole Oceanographic Institution. “Freshwater would essentially float on the surface and cap off mixing and heat transfer,” he explains, which ultimately speeds up the process of warming—producing an exponential increase in the rate of ice-edge retreat.

One of the potential scenarios with rising temperatures, says Hofmann, is that the Antarctic Circumpolar Current will move farther away from the continental shelf along the peninsula. “That would greatly diminish the supply of CDW, and change the physical and biological structure of the shelf,” she says. Alternatively, the Antarctic Circumpolar Current would get pinned against the continental shelf. “If CDW is continually pumped onto the shelf, then it probably would warm up the whole shelf water above freezing,” says Hofmann. And that, she says, could lead to a collapse of the sea ice.

## Retreating Sea Ice and Krill Declines

In November 2004, the most comprehensive study to date of krill distribution and abundance in the Southern Ocean reported a catastrophic drop in krill numbers. Angus Atkinson, a marine biologist with the British Antarctic Survey, led the study. “We pulled together all the net samples we could lay our hands on that had been obtained in the Southern Ocean over the last 80 years,” Atkinson says, analyzing nearly 12,000 krill summer net hauls taken from 1926–1939 and from 1976–2003.

“The Southern Ocean is an enormous area, and at least half of krill stocks were in this comparatively narrow sector between South Africa and the Antarctic Peninsula,” he says. His team found a positive correlation between winter sea ice cover and the abundance of krill the following summer. There's evidence of a general decline in winter sea ice extent and duration, Atkinson says, and of a general decline in krill populations—down 80% over the past 30 years—over the entire southwest Atlantic sector.

Though krill populations showed big fluctuations in the early years, their average numbers were higher over a longer period, explains Volker Siegel, a krill biologist with the Sea Fisheries Research Institute in Hamburg, Germany, who worked with Atkinson. “Where in the early days you might have 100 krill per square meter on average—with fluctuations between, say 20 and 300—nowadays you might see 20 per square meter, which goes from 50 to five individuals per square meter.”

Neither Atkinson nor Siegel can say for sure what's causing the decline, but both say the winter sea ice is clearly playing some role. Krill live about five to six years. During the breeding season, from December to March, embryos are released in the upper water column, and the larvae hatch at depths ranging from 400 meters to 1,500 meters, unlike many fish and other invertebrate larvae, which hatch at the surface. Larvae then have to swim up through the water column to reach the sea surface. Unlike adults, krill larvae don't have enough body fat to carry them through food shortages. “They'll starve if they have to rely on water-column food distributions, and that's where the sea ice comes in,” says Atkinson. “The ice may also shelter them from predators, but one way or another, ice in the winter is good for young krill.”

“We've got to find out what's causing these changes and then we can start to predict what's going to happen with future scenarios of climate change,” Atkinson says. “The other thing we've got to do is look at alternative things which might be affecting krill. We might find there are other things declining as well as sea ice, such as their food, or there might be a change in the fertilization of the waters.”

Accompanying the drop in krill abundance, Atkinson and Siegel found an increase in salps, transparent jelly-like creatures that typically inhabit warmer waters than krill. Expanding into the warmer waters, salp populations are increasing in the southern part of their range and replacing the krill. Most krill-dependent predators do not eat salp.

## Krill Declines Ripple up the Food Chain

At a hearing on climate change impacts before the United States Senate Committee on Commerce, Science, and Transportation in May 2004, LTER's Fraser testified that the western Antarctic Peninsula's cold, dry polar marine ecosystem is gradually being replaced with a warm, moist maritime climate. While all the major components of the food web are responding to these changes, Fraser said, the clearest evidence comes from studies of two especially sensitive indicators of climate change: krill and penguins. “Trends in penguin populations provided some of the first evidence that sea ice conditions in some areas were deteriorating in response to climate warming,” Fraser told the senators.

**Figure 2:**
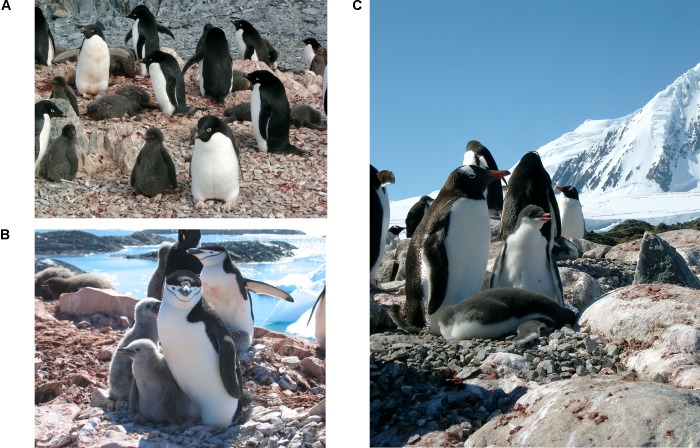
Antarctic Penguins Show Striking Contrasts in Their Relationship to the Sea Ice (A) Ice-dependent Adélie penguins, at their nesting grounds on Anvers Island, have lost 10,000 breeding pairs since 1975. (B) Ice-avoiding chinstrap penguins at Anvers Island are increasing in number and range. (C) Breeding gentoo penguins—an ice-avoiding species that has not inhabited Anvers Island sites for at least 800 years—are turning up at Palmer Station. (Images: Donna Fraser)

This evidence came from Fraser's own studies, over the past 30 years, of three Antarctic penguin species that share similar life histories (including a penchant for krill) but show striking contrasts in their relationship to the sea ice (Figure 2). For Adélie (Pygoscelis adeliae) penguins, the presence of sea ice is absolutely essential for survival. Chinstrap (P. antarctica) and gentoo (P. papua) penguins, on the other hand, require the absence of sea ice. “Adélie penguins have experienced a nearly 70% decrease in their populations at our study sites on Anvers Island [Palmer Station] in the western Antarctic Peninsula over the last 30 years,” says Fraser, “and there's evidence that other krill-dependent predators are beginning to decrease.”

That's a loss of 10,000 breeding pairs since 1975. The major factors underlying this precipitous decline, Fraser says, are retreating sea ice and increasing snowfall. (The loss of sea ice increases the flow of water vapor from the open ocean to the atmosphere, increasing precipitation.) “When sea ice forms,” he explains, “it covers these regions of high production and the birds are just able to plop into the water into very good feeding areas.” With a life history accustomed to the formation of sea ice at critical points in their life cycle, Adélies are finding themselves faced with an unpredictable sea ice cycle that outpaces their ability to adapt. “The birds just don't have the sea ice when they need it,” Fraser says. As if losing critical winter habitat weren't bad enough, Adélies must also contend with the effects of increased snowfall. When the snow melts in the spring, it's flooding their nesting areas and drowning their eggs and chicks.

The population trends for “ice-avoiding” chinstraps and gentoos are quite different. Both species are increasing their populations and beginning to replace Adélie penguins across a broad range in the western Antarctic Peninsula. “We're seeing breeding gentoo penguins at Palmer Station,” Fraser says, with a trace of astonishment. “The paleoecological record does not show that species at our study sites in the last 800 years.”

Though chinstraps and gentoos also depend on krill, they've dealt with krill declines by eating more fish and squid (which, not incidentally, also eat krill). Fraser believes this dietary flexibility, along with the increased availability of open water and a late breeding schedule (which protects their eggs and chicks from spring snow meltwater), largely explain their range extensions.

“Adélies don't seem capable of adjusting anything about their life history,” says Fraser. “They're hard-wired to their breeding area, returning to an area year after year after year, even though conditions are deteriorating.” And so, their numbers continue to plummet as more chicks perish. Another ice-dependent, krill-eating penguin species, the emperor (Aptenodytes forsteri), is not faring any better. “We have just one emperor colony in our study region, and it's decreasing very fast,” Fraser says. “It's gone down from 300 breeding pairs to nine. They're on the verge of extinction in our study region.”

## A Question of Sustainability

A major issue in devising strategies to protect krill populations concerns the impact of krill fisheries. Since drastic declines have occurred in the absence of heavy fishing, it's especially important to establish the population dynamics of Antarctic krill. Siegel works with the Convention for the Conservation of Antarctic Marine Living Resources (CCAMLR) to develop sustainable fishing regulations. Based on a point estimate of 44 million metric tons of krill in the southwest Atlantic sector in 2000, CCAMLR calculated a potential yield of 4 million tons, far above the average 100,000-metric-ton catch today. Siegel believes the fisheries aren't posing a significant threat to krill stocks at this point, but says much remains to be learned about krill population structure.

Gallager is not so sure about the impact of krill fishing. Some krill fisheries operate near the island of South Georgia, east of the Falkland Islands. “We know that these populations are not self-sustaining, but require recruiting of adults from other locations, and probably from regions along the western peninsula of Antarctica,” says Gallager. “If we go in and fish out the populations that are not self-seeding, then they could be entirely wiped out.” That's why it's crucial to know which populations, if any, are self-sustaining, he says. “And we don't know a lot about that at all.”

Toward that end, Gallager is working on an under-ice “zooplankton observatory” that would sit on the bottom of the ocean floor and continuously release a flotation package—“once an hour, for hopefully the next ten years”—outfitted with sonar and optical sensors up to the surface, then winch itself back down again. The plan is to deploy the observatory off Palmer Station in May 2006. The hope is that data gathered on over-wintering larval krill will shed light on the factors influencing krill survival over the long term. “It may be that this coupling between larval krill and ice is actually underpinning the entire question of krill population dynamics,” says Gallager—in which case, understanding how the ice moves relative to the water currents and wind shear over the top will be critical.

Many other aspects of krill biology remain obscure as well. Biologists still don't understand the precise mechanisms required to enhance larval growth, reproduction, and recruitment (replenishing populations with new individuals), or how temperature fluctuations affect metabolism and larval growth rate. Robust tests of long-held theories of how the under-ice habitat sustains krill larvae require much more quantitative data—over time and over a wide scale—on larval abundance, distribution, and foraging behavior. But netting krill is not easy. Larvae tend to wedge themselves into nooks and crannies, defying divers' attempts to nab them while protecting their expensive nets— loaded with even more expensive electronic gear—from the ice.

Whatever is behind the correlation between sea ice decline and krill declines, the future of the Antarctic ecosystem hangs in the balance. A 2001 report by the Intergovernmental Panel on Climate Change predicted that the Antarctic Peninsula will experience some of the largest, most rapid climate changes on earth. If these trends of rising temperatures and decreasing sea ice continue, says LTER's Fraser, “what we are going to see in the next ten, 20, 30 years is a system that is completely different from the one that exists now. Adélies will become regionally extinct.”

Concluding his testimony on climate change impacts, Fraser warned the US Senate committee that if future warming continues and the cycle of heavy ice years exceeds the life span of krill, the species will face a reproductive crisis. And that, he said, “will have catastrophic consequences to the integrity of this marine ecosystem.”

In 1912, for the sake of a few emperor penguin eggs, Apsley Cherry-Garrard and two members of Scott's ill-fated polar expedition endured what Cherry-Garrard called “extremity of suffering” from which only “madness or death may give relief.” The group believed the eggs might prove that the penguins were the missing link between “birds and the reptiles from which birds have sprung.”

Our understanding has advanced light years since then, rendering such notions nearly quaint. Cherry-Garrard and his companions thought the forbidding Antarctic landscape immune to human assaults. Today, with this notion, too, proven false, one wonders if the damage can be reversed. In Atkinson's diplomatic phrase, “there are political issues involved” where global warming is concerned. But the clock is ticking. If the Antarctic ecosystem collapses, it won't be because scientists were off on a misguided search for penguin eggs.

**Figure 3:**
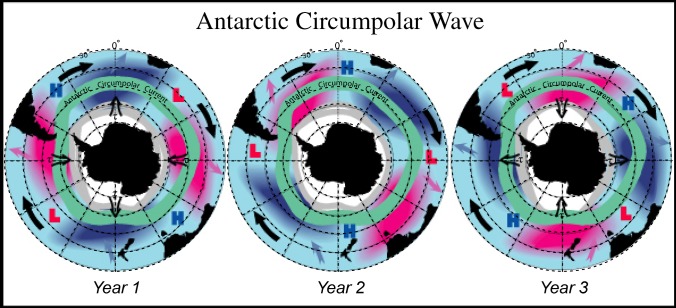
Antarctic Circumpolar Wave In 1996, oceanographers Warren White and Ray Peterson identified significant inter-annual variations in the atmospheric pressure at sea level, wind stress, sea surface temperature, and sea-ice extent over the Southern Ocean. They called this system of coupled anomalies the Antarctic Circumpolar Wave. This simplified schematic summarizes the inter-annual variations in sea surface temperature (red, warm; and blue, cold), atmospheric sea-level pressure (bold H and L), meridional wind stress (denoted by τ), and sea ice extent (gray lines), together with the mean course of the Antarctic Circumpolar Current (green). Heavy black arrows depict the general eastward motion of anomalies, while other arrows indicate communications between the circumpolar current and the more northerly subtropical gyres. (Image: Warren White, http://jedac.ucsd.edu/ACW/index_research.html)

## Abbreviations

CCAMLR: Convention for the Conservation of Antarctic Marine Living Resources
CDW: Circumpolar Deep Water
LTER: Palmer Long Term Ecological Research program
