# Correction: As the Antarctic Ice Pack Recedes, a Fragile Ecosystem Hangs in the Balance

**DOI:** 10.1371/journal.pbio.0030224

**Published:** 2005-06-14

**Authors:** Liza Gross

In *PLoS Biology*, volume 3, issue 4, DOI: 10.1371/journal.pbio.0030127

The figure credits for Figures 1 and 2 are missing from the PDF versions of the article. For Figure 1, images are courtesy of Langdon Quetin and Robin Ross, researchers at the Marine Science Institute, University of California, Santa Barbara, and funded by the Office of Polar Programs, National Science Foundation. For Figure 2, images are courtesy of Donna Fraser.

